# Global research trends and hotspots of artificial intelligence research in spinal cord neural injury and restoration—a bibliometrics and visualization analysis

**DOI:** 10.3389/fneur.2024.1361235

**Published:** 2024-04-02

**Authors:** Guangyi Tao, Shun Yang, Junjie Xu, Linzi Wang, Bin Yang

**Affiliations:** ^1^College of Orthopedics and Traumatology, Henan University of Traditional Chinese Medicine, Zhengzhou, China; ^2^Department of Pain, Henan Provincial Hospital of Traditional Chinese Medicine/The Second Affiliated Hospital of Henan University of Traditional Chinese Medicine, Zhengzhou, China

**Keywords:** artificial intelligence, neural regeneration, spinal cord injury, rehabilitation robot, brain-computer Interface, neuroelectrical stimulation, convolutional neural network, deep learning

## Abstract

**Background:**

Artificial intelligence (AI) technology has made breakthroughs in spinal cord neural injury and restoration in recent years. It has a positive impact on clinical treatment. This study explores AI research’s progress and hotspots in spinal cord neural injury and restoration. It also analyzes research shortcomings related to this area and proposes potential solutions.

**Methods:**

We used CiteSpace 6.1.R6 and VOSviewer 1.6.19 to research WOS articles on AI research in spinal cord neural injury and restoration.

**Results:**

A total of 1,502 articles were screened, in which the United States dominated; Kadone, Hideki (13 articles, University of Tsukuba, JAPAN) was the author with the highest number of publications; ARCH PHYS MED REHAB (IF = 4.3) was the most cited journal, and topics included molecular biology, immunology, neurology, sports, among other related areas.

**Conclusion:**

We pinpointed three research hotspots for AI research in spinal cord neural injury and restoration: (1) intelligent robots and limb exoskeletons to assist rehabilitation training; (2) brain-computer interfaces; and (3) neuromodulation and noninvasive electrical stimulation. In addition, many new hotspots were discussed: (1) starting with image segmentation models based on convolutional neural networks; (2) the use of AI to fabricate polymeric biomaterials to provide the microenvironment required for neural stem cell-derived neural network tissues; (3) AI survival prediction tools, and transcription factor regulatory networks in the field of genetics were discussed. Although AI research in spinal cord neural injury and restoration has many benefits, the technology has several limitations (data and ethical issues). The data-gathering problem should be addressed in future research, which requires a significant sample of quality clinical data to build valid AI models. At the same time, research on genomics and other mechanisms in this field is fragile. In the future, machine learning techniques, such as AI survival prediction tools and transcription factor regulatory networks, can be utilized for studies related to the up-regulation of regeneration-related genes and the production of structural proteins for axonal growth.

## 1 Introduction

Spinal cord neural injury is a neurological injury due to direct or indirect factors, characterized by motor and perceptual dysfunction, abnormal muscle tone, and various other pathological feedbacks in the corresponding injured segment (1, 2). Currently, applied treatments in medicine usually fail to meet expectations, and research focuses mainly on using drugs, cellular therapies, and tissue engineering.

Artificial Intelligence (AI) is a generic term that implies the use of computers to model intelligent behavior with minimal human intervention, and it is described as the science and engineering of building intelligent machines. There are two main branches of AI in medicine: virtual and physical. The virtual branch consists of informatics methods ranging from deep learning information management to control of health management systems, including electronic health records and active guidance of physicians in treatment decisions. The physical branch is represented by robots used to help patients or surgeons. Artificial intelligence has recently emerged to analyze and manipulate nerve reproduction and recovery information. AI can rate the extent of neural plastination and efficacy of nerve stem cells, and studies of neural injury and restoration could also offer valuable data resources for AI (3). Meanwhile, AI can also help translate nerve signaling and control machine exoskeletons (4). In addition, artificial intelligence can also discern which gene and signaling pathway is critical for nerve recovery (5).

Therefore, AI systems and research on spinal cord neural injury and restoration can mutually reinforce each other and drive medical innovation. We used popular bibliometric software (CiteSpace and VOSviewer) to visualize and analyze the development history and research hotspots of AI research in spinal cord neural injury and restoration to analyze research shortcomings related to this area and propose potential solutions.

## 2 Data and methods

### 2.1 Retrieval strategy

**Figure 1:**
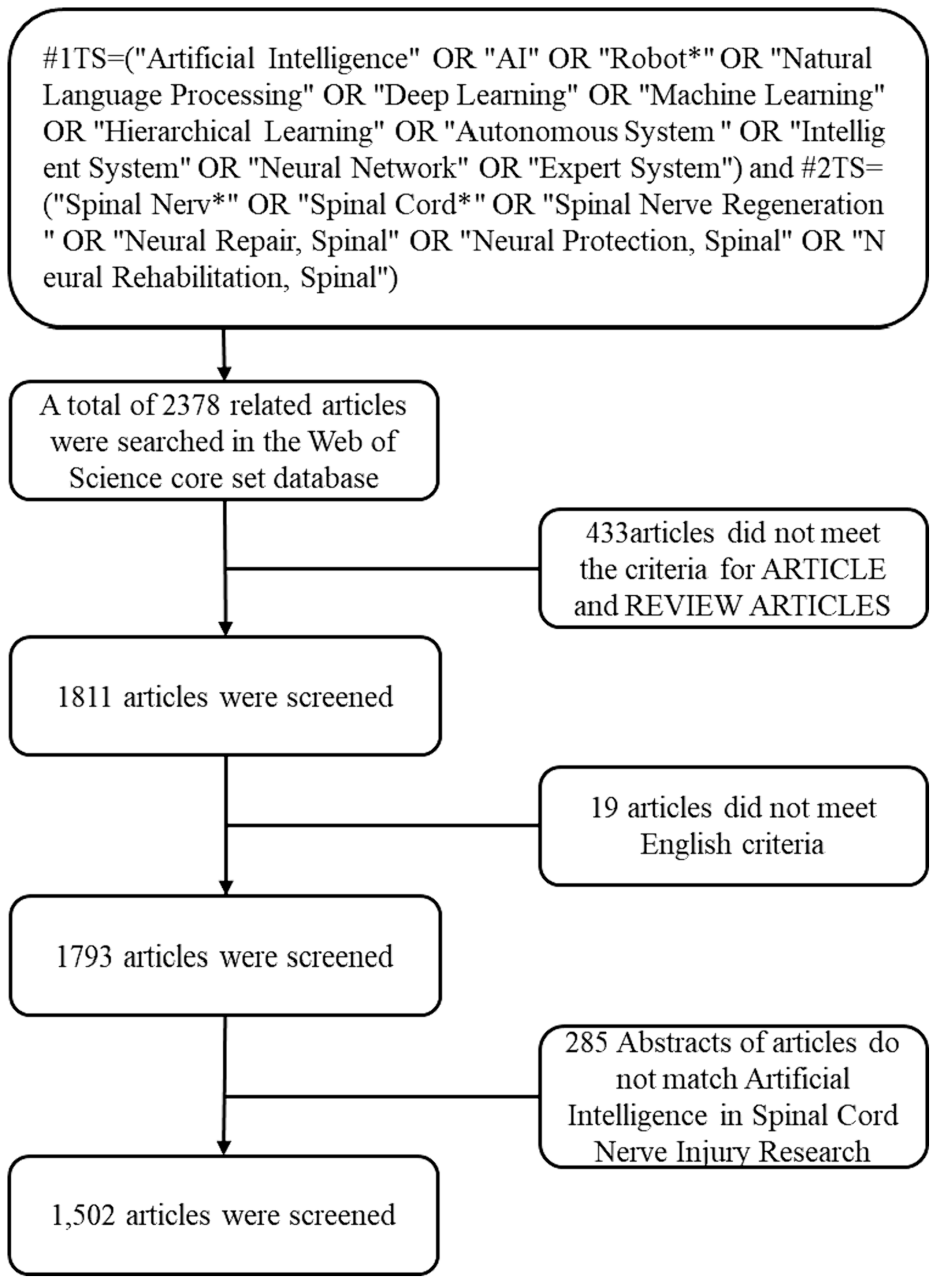
Flow chart of the literature search strategy and selection in this study.

We searched the Web of Science (WOS) core collection for literature on AI research in spinal cord neural injury and restoration. A literature search was completed on March 5, 2024. The WOS retrieval formula (6) was #1TS = (“Artificial Intelligence” OR “AI” OR “Robot*” OR “Natural Language Processing” OR “Deep Learning” OR “Machine Learning” OR “Hierarchical Learning” OR “Autonomous System “OR “Intelligent System”); #2TS = (“Spinal Nerv*” OR “Spinal Cord*” OR “Spinal Nerve Regeneration” OR “Neural Repair, Spinal” OR “Neural Protection, Spinal” OR “Neural Rehabilitation, Spinal”); #1AND #2 (see Figure 1). Select article, review, and English.

### 2.2 Data collection

After the preliminary data retrieval, two researchers (T Gy and Y Bin) screened all manuscripts separately to ensure they were relevant to the theme of this study (see Figure 1). The final results were exported as a “plain text file,” with “Full Record and Cited References” selected as the record content and stored in download_*.txt format.

### 2.3 Parameter settings and critical observations

Parameterization of VOSviewer 1.6.19: Inter-country publication analysis (up to a minimum number of 24 papers) and keyword clustering analysis were performed using VOSviewer software.

Parameterization of CiteSpace 6.1.R6: The time parameter was set from January 2004 to March 2024, 1 year as the time zone, Top *N* = 50, cropping was Pathfinder, Pruning sliced network and Pruning the merged networks, and the other settings were kept as default; Select keywords, literature, and journals for co-occurrence analyses and co-citation analyses.

1. Conducting co-citation analyses of papers to define main research directions and hotspots. The following aspects are proposed to construct the co-citation graph: take the papers as nodes, cite frequency as the node size, link the literature with the co-citation relationship, and perform cluster analysis.
2. Creating a keyword co-occurrence graph to analyze the emergent words.
3. Creating the co-citation analysis graph of “hot” journals and studying its distribution in various disciplines.
4. Creating a two-plot superimposed journal map showing, among other things, citation trajectories and focus drift in the field.

## 3 Results

### 3.1 Trend analysis of global publication output

**Figure 2:**
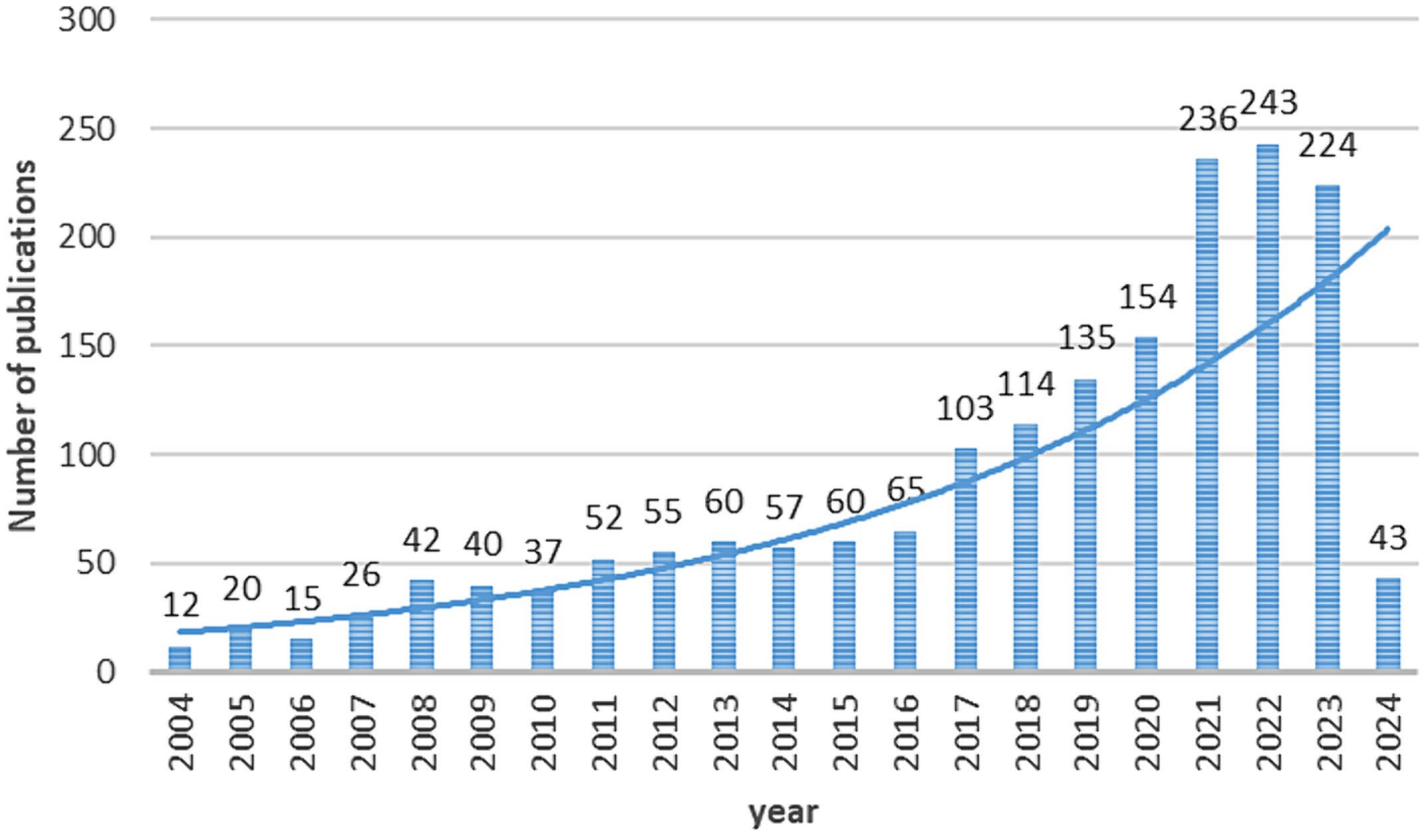
Publication trends for AI research in spinal cord neural injury and restoration. The curve represents a continuous increase in the trend of publications from 2004 to 2024.

Based on the selection procedure, 1,502 papers on AI research in spinal cord neural injury and restoration were collected from the WoS database. Only 12 articles were published in this field in 2004, and no relevant literature was published before 2004. On the whole, the number of published papers is on the rise, indicating that the attempts and explorations made by scholars for AI research in spinal cord neural injury and restoration are gradually increasing, and its research value has been emphasized by many researchers in the academic community, see Figure 2.

### 3.2 Country/region analysis and author analysis

**Figure 3:**
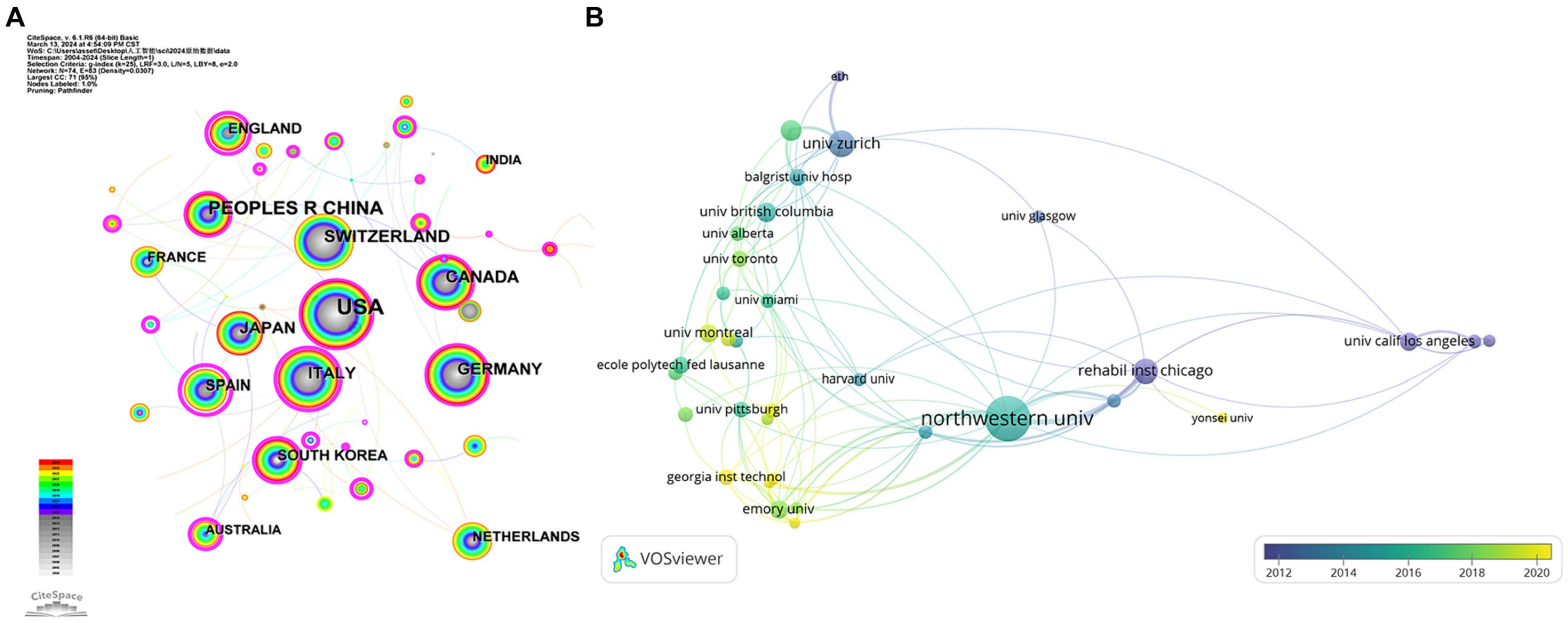
National analysis map for AI research in spinal cord neural injury and restoration. **(A)** Country analysis graph. **(B)** Institution analysis graph. Each node represents a country (or institution), and its size represents the number of publications; the thickness of the lines means the intensity of cooperation between countries (or institution); the thicker the strings, the higher the intensity of collaboration. Different colors represent different times.

The VOSviewer 1.6.19 result indicates that 20 nations have at least 19 publications on the research topic (see Figure 3). As can be seen in Figure 3, there is a growing global enthusiasm for AI research in spinal cord neural injury and restoration, with the highest number of papers published in Asia and America. However, as a whole, the strength of the connection between countries is relatively fragmented, indicating that international cooperation still needs to be strengthened.

The analysis results of the authors and institutions are shown in Table 1. Among them, Northwestern Univ and Univ Zurich have higher centrality, indicating that they have close connections with other institutions and frequently cooperate in conducting research and publishing articles.

**Table 1:** Information table of included literature.

	Classification	具体信息
1	Time of inclusion	2004.1–2024.3
2	Literature Topic	artificial intelligence assisted repair of spinal cord neural injury
3	Type of Literature	article、review
4	Country of Literature	USA(534)、CHINA(194)、SWITZERLAND(130)、CANADA(125)
5	Journal considered	J NEUROENG REHABIL(77)、IEEE T NEUR SYS REH(46)、J SPINAL CORD MED (7)、NEUROREHABILITATION (8)

### 3.3 Analysis of research hotspots and “hot” journals based on journal citations

#### 3.3.1 Analysis of journal co-citation bursts

**Figure 4:**
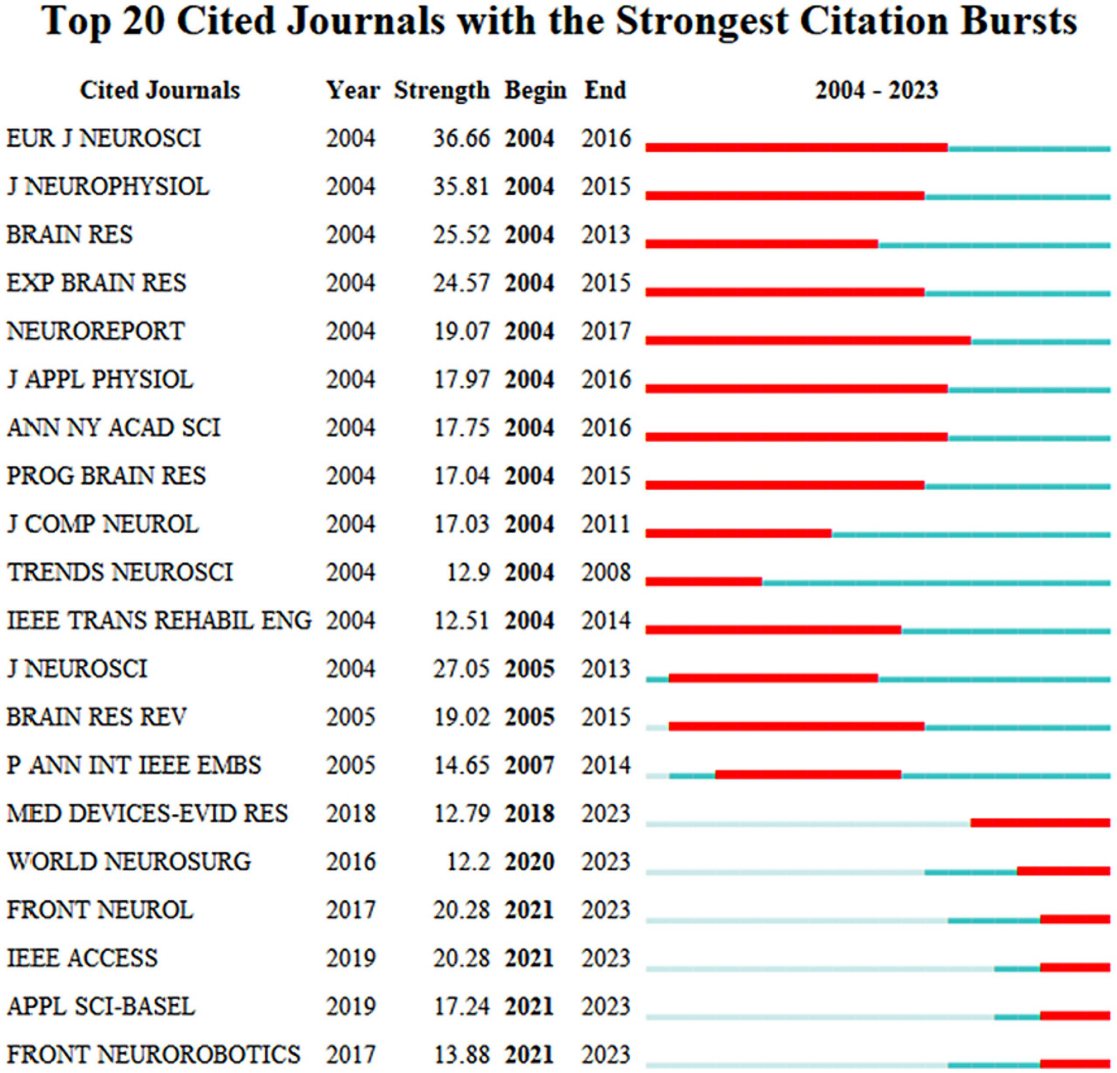
The 20 most cited journals for AI research in spinal cord neural injury and restoration. Outbreak journals have been heavily cited during a specific period. This chart lists the 20 outbreak journals identified in this research area from papers published from 2004 to 2024. The red box indicates the year in which the outbreak began, “year” means the earliest year of appearance, “Strength” is the number of references, “Begin” and “End” represent the beginning and end of the burst.

NEUROREPORT has paid the most attention to AI-assisted repair of spinal cord neural injury and restoration research and has paid attention to related hotspots for a more extended period (2004–2017). In the last 5 years (2019–2023), MED DEVICES-EVID RES, WORLD NEUROSURG, FRONT NEUROL, and IEEE ACCESS are the major journals focusing on the field of AI-assisted repair of spinal cord neural injury and restoration (see Figure 4). ARCH PHYS MED REHAB is the most cited journal (see Table 2).

**Table 2:** Top 10 researchers with the most publications on AI research in spinal cord neural injury and restoration.

Rank	Researcher	Number of publications	Affiliated country
1	Kadone, Hideki	13	JAPAN
2	Jayaraman, Arun	12	USA
3	Riener, Robert	11	SWITZERLAND
4	Yamazaki, Masashi	10	JAPAN
5	Sankai, Yoshiyuki	9	JAPAN
6	Zeng, Xiang	9	CHINA
7	Edgerton, V Reggie	9	USA
8	Gil-agudo, Angel	9	SPAIN
9	Marushima, Aiki	8	JAPAN
10	Cohen-added, Julien	8	CANADA

#### 3.3.2 Journal biplot overlay analysis

**Figure 5:**
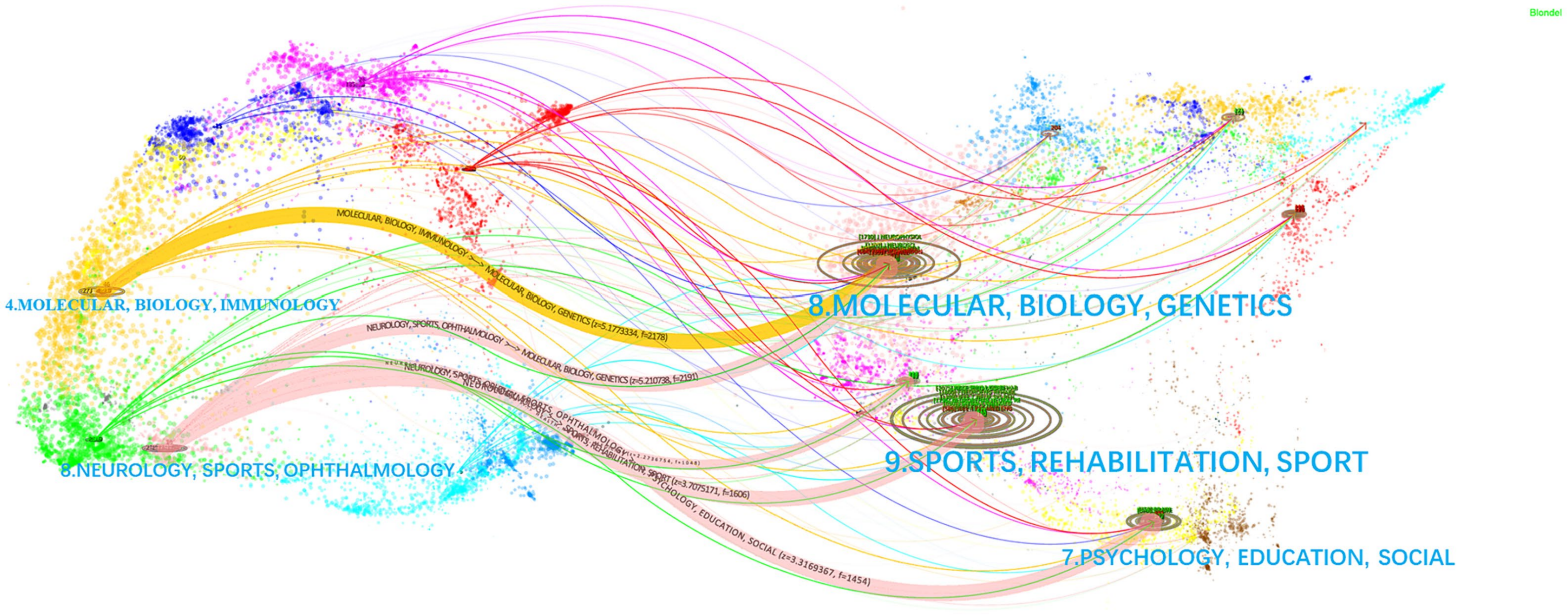
Two-map overlay of cited/cited journals for AI research in spinal cord neural injury and restoration. Each publication is added to two interrelated but different global science maps, with the citing publication on the left and the cited publication on the right. Each point on this map is an article with a corresponding magazine. The curves are citation links representing citation paths. The ellipses represent the citation frequency of the clusters. The data for the five curves in the figure are (z = 5.177, *f* = 2,178), (z = 5.211, f = 2,191), (z = 2.274, *f* = 1,048), (z = 3.708, f = 1,606), and (z = 3.317, f = 1,454). f: frequency of citations from left citing journals to right cited journals, z: normalization to the value of f. z and f represent the closeness and importance of the linkage.

Based on CiteSpace’s research base data, Journal Citation Reports (JCR) 2011 data were analyzed using the Blondel algorithm for journal biplot overlay analysis of the literature in this area (see Figure 5), with the citing journals on the left and the cited journals on the right—the citing journal concentrated on MOLECULAR, BIOLOGY, IMMUNOLOGY; or NEUROLOGY, SPORTS, OPHTHALMOLOGY. The most significant direction of cited journals was SPORTS, REHABILITATION. NEUROLOGY, SPORTS, OPHTHALMOLOGY, and MOLECULAR BIOLOGY GENETICS are most strongly associated with the journal and are the “hot” areas for AI-assisted repair of spinal cord neural injury and restoration (z = 5.211, *f* = 219).

### 3.4 Keyword clustering analysis and burst analysis

**Figure 6:**
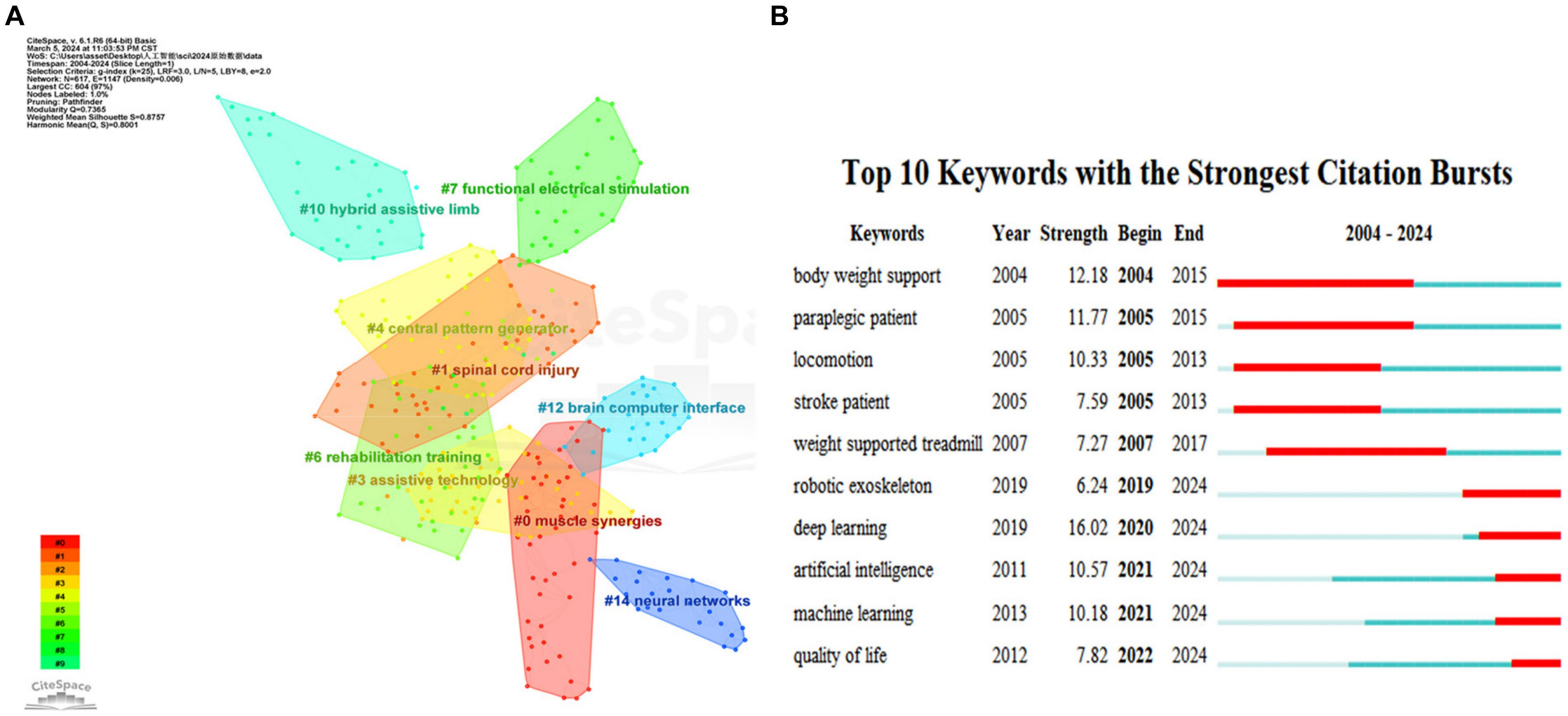
Keyword network diagram for AI research in spinal cord neural injury and restoration. **(A)** Keyword clustering network graph. Each node represents a keyword; The colors represent the year the clusters began to appear (Notes: #0 muscle synergies, #1 spinal cord injury, #3 assistive technology, #4 central pattern generator, #6 rehabilitation training, #7 functional electrical stimulation, #10 hybrid assistive limb, #12 brain-computer interface, #14 neural networks). **(B)** Keyword outbreak analysis chart. The red box represents the year the burst started, “year” means the earliest year of occurrence, and “strength” is the number of citations.

Keyword clustering analysis was performed using CiteSpace 6.1.R6 software (see Figure 6A), including nine main clusters: #0 Muscle synergy, #1 spinal cord injury, #3 assistive technology, #4 central pattern generator, #6 rehabilitation training, #7 functional electrical stimulation, #10 hybrid assistive limbs, #12 brain-computer interface, and #14 neural networks. Three main research directions for AI research in spinal cord neural injury and restoration were identified: (1) research on assistive exoskeletons and motor rehabilitation (#0, #3, #6, #10); (2) research on brain-computer interfaces (#4, #12, #14); and (3) research on functional electrical nerve stimulation (#7).

Keyword bursting was performed using CiteSpace 6.1.R6 software. It consisted of two main phases: (1) the 2004-2017 focus direction was less intelligent robotic exoskeletons assisting weight support and locomotion in paraplegic patients and (2) the 2021-2024 focus direction was deep learning algorithms to enhance artificial intelligence (see Figure 6B).

### 3.5 Literature co-citation analysis

#### 3.5.1 Literature co-citation cluster analysis

**Figure 7:**
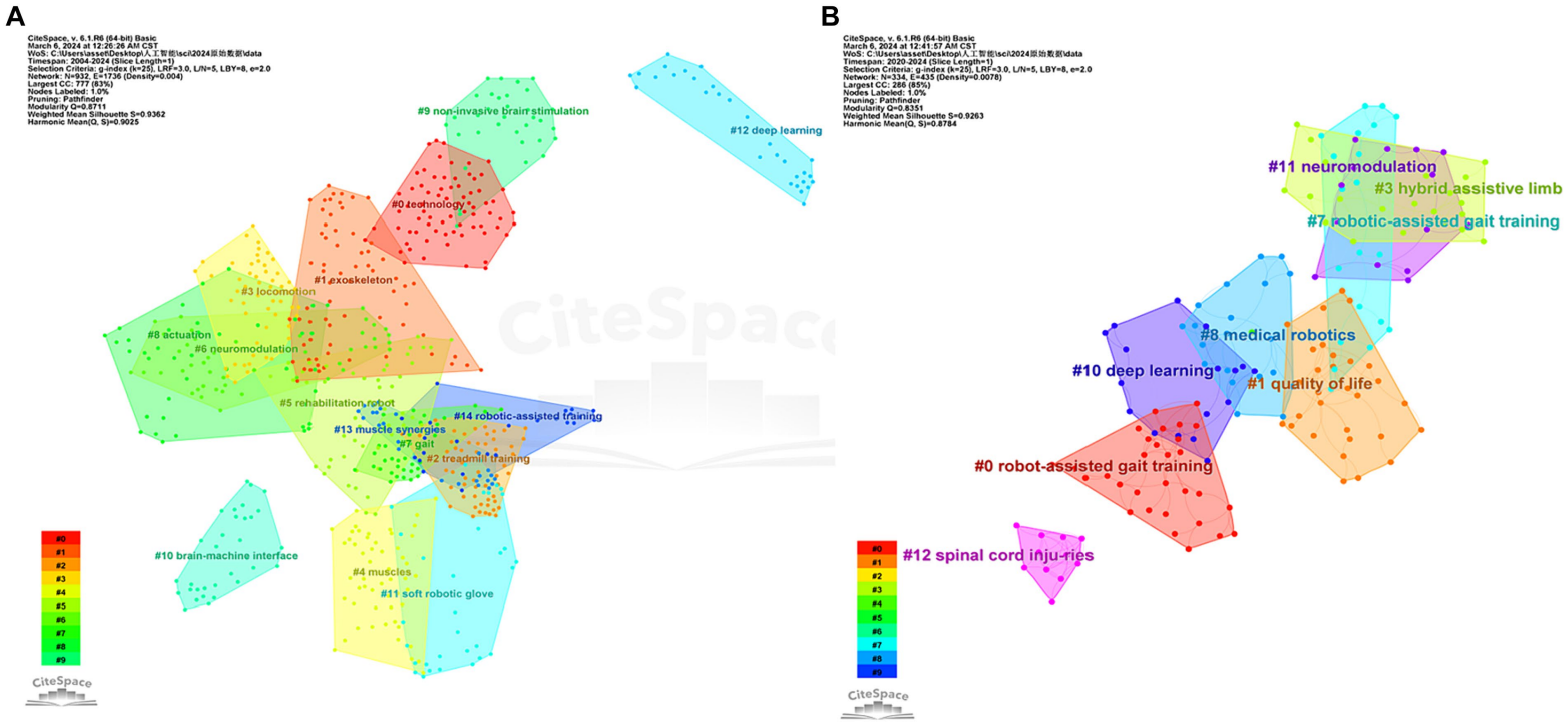
Co-citation clustering of literature on AI research in spinal cord neural injury and restoration. **(A)** Literature co-citation clustering diagram for AI in spinal cord neural injury and restoration; **(B)** Literature co-citation clustering diagram for research hotspots in the last 5 years (2020–2024). The clusters’ color represents the year of the first co-citation relationship, The nodes represent the cited publications, and their size represents the number of times.

We obtained 25 clusters, including the 15 most significant clusters in the literature co-citation network (Figure 7A with Table 3).

**Table 3:** Top 10 most cited journals for AI research in spinal cord neural injury and restoration.

Rank	Journal	Impact factor	Citation count	Centrality	Rank
1	ARCH PHYS MED REHAB	4.3	Q1	658	0
2	SPINAL CORD	2.2	Q2	612	0.03
3	J NEUROENG REHABIL	5.1	Q1	603	0.01
4	IEEE T NEUR SYS REH	4.9	Q1	485	0
5	J SPINAL CORD MED	1.7	Q4	453	0.08
6	NEUROREHAB NEURAL RE	4.2	Q1	447	0.06
7	J NEUROPHYSIOL	2.5	Q3	407	0
8	PHYS THER	3.8	Q1	402	0
9	PLOS ONE	3.7	Q2	391	0.01
10	J NEUROSCI	5.3	Q1	380	0.02

Clusters #0, #1, #2, #7, #8: Technology, Exoskeletons, Gait, treadmill training and Actuation. These clusters are all focused on machine exoskeletons to assist patients with rehabilitation exercises, and the main cited articles are Evans et al. (9) and Sanchez et al. (10). In recent years, robotic motion exoskeletons have provided standing and walking opportunities for people with spinal cord injury and considerable solutions for gait assistance and rehabilitation. The field focuses on actuation, structure, and interface connectivity components.

Clusters #3, #4, #5, #14: Rehabilitation Robot-Assisted Gait Training. The principal cited articles for these two clusters are Banala et al. (11) and Fang et al. (12). Gait training is critical for promoting neuromuscular plasticity, which is necessary to improve functional walking ability. Robot-assisted gait training was developed for spinal cord injury patients using active leg exoskeletons and force field controllers, which effectively apply force at the subject’s ankle through actuators on the hip and knee joints for rehabilitation.

Clusters #9, #10: Brain-Computer Interfaces and Noninvasive Brain Stimulation. The primary cited article in this cluster is Collinger et al. (13). Upper limb paralysis or amputation results in the loss of the ability to grasp., manipulate, and carry objects in the upper limbs. These functions are critical for activities of daily living. Brain-computer interfaces can provide a solution for restoring many of these lost functions. In this paper, two 96-channel intracortical microelectrodes were implanted in a patient’s motor cortex to test that quadriplegic patients can use this brain-computer interface to rapidly achieve neural control of a high-performance prosthesis.

Cluster #6: neuromodulation. The primary cited article in this cluster is Angeli et al. (14), which demonstrated that neuromodulation of spinal circuits by epidural stimulation enables wholly paralyzed patients to regain relatively fine autonomous control over paralyzed muscles. That neuromodulation of excitatory subthreshold motor states in the lumbosacral spinal cord network is the key to restoring conscious movement in individuals diagnosed with complete leg paralysis. A novel intervention strategy was discovered that significantly impacts the recovery of voluntary action in completely paralyzed individuals even years after injury.

#### 3.5.2 Cluster analysis of co-cited literature on research hotspots in the last 5 years

We obtained the nine most significant clusters in the literature co-citation network (Figure 7B with Table 4).

**Table 4:** AI research in spinal cord neurological injury and repair nine most representative literature co-citation clusters.

Cluster-ID	Size	Silhouette	Label LLR
#0	73	0.923	Technology
#1	67	0.93	Exoskeleton
#2	57	0.927	Treadmill training
#3	56	0.987	Locomotion
#4	54	0.855	Muscles
#5	53	0.824	Rehabilitation robot
#6	46	0.967	Neuromodulation
#7	40	0.962	Gait
#8	40	0.957	Actuation
#9	35	0.971	Non-invasive brain stimulation
#10	32	0.934	Brain-machine interface
#11	32	0.966	Soft robotic glove
#12	30	1	Deep learning
#13	26	0.966	Muscle synergies
#14	25	0.936	Robotic-assisted training

The silhouettes are the average contour values of the clusters (Tables 3 and this table). Generally, groups with silhouette scores > 0.5 were accepted, and groups with silhouette scores > 0.7 had good clustering performances. The size represents the number of items in each group, and labels represent the clusters using the LLR algorithm.

Clusters #0, #3, #7, #8: Robotic Motion Exoskeleton Assisted Movement and Rehabilitation. Both groups are focused on machine exoskeletons, and the main cited articles are Fang et al. (12) and Sanchez et al. (10). The field focuses on actuation, structure, and interface connections.

Cluster #1, #10: Brain-computer interface technologies. The principal cited article in this cluster is Ajiboye et al. (15), which allows for the restoration of limb movement in patients with chronic quadriplegia through coordinated electrical stimulation of the surrounding muscles and nerves (also known as functional electrical stimulation); the patient’s cortical signals can be used to direct limb movement through an implanted practical electrical stimulation component and an intracortical brain-computer interface. This is the first co-implanted functional electrical stimulation + intracortical brain-computer interface neuroprosthesis and represents a significant advancement in the clinical feasibility of neuroprostheses.

Cluster #11, #12: Overview of Neuromodulation and Electrical Stimulation. The primary cited article in this cluster is Gill et al. (16), where spinal sensory-motor networks that are functionally disconnected from the brain as a result of spinal cord injury can be facilitated by epidural electrical stimulation to encourage the return of robust, coordinated motor activity in paralyzed patients. Dynamic task training in the presence of epidural electrical stimulation is referred to as multimodal rehabilitation in this study. This article is the first report of such multimodal rehabilitation in patients with sensory and motor loss of the lower extremities due to spinal cord injury.

#### 3.5.3 Hot spot analysis of co-cited literature

The cited literature of all nodes was ranked according to the number of co-citations, and the 10 articles with the highest number of co-citations are shown in Table 5. The main hotspots are AI exoskeleton and robot-assisted gait training (Table 6).

**Table 5:** The four most representative literature co-citation clusters in research hotspots in the last 5 years (2019–2023).

Cluster-ID	Size	Silhouette	Label (LLR)
#0	64	0.863	Robot-assisted gait training
#1	43	0.974	Quality of life
#3	36	0.849	Hybrid assistive limb
#7	30	0.974	Robotic-assisted gait training
#8	27	0.934	Medical robotics
#10	26	0.962	Deep learning
#11	23	0.85	Neuromodulation
#12	21	0.894	Spinal cord injuries
#15	19	0.947	Robot

**Table 6:** Top 10 cited publications on AI research in spinal cord neural injury and restoration.

Citations	Cluster-ID	Label LLR	Article topic	Citation count
Miller, 2016 (17)	#10	Flexotendon glove-iii	Dynamic exoskeleton + meta-analysis	74
Esquenazi, 2012 (18)	#3	Potential use	ReWalk dynamic exoskeleton + complete thoracic spinal cord injury	67
Louie, 2015 (19)	#0	Robotic locomotor exoskeleton	Powered robotic exoskeleton + assisted walking	58
Wirz, 2005 (20)	#1	Robotics-assisted treadmill exercise	Robot-assisted driven gait orthosis + locomotion training	55
Baunsgaard, 2018 (21)	#10	Flexotendon glove-iii	Robotic exoskeleton + gait training	50
Nam, 2017 (22)	#12	Gait rehabilitation	Robot-assisted gait training + neurophysiological mechanisms	50
Kozlowski, 2015 (23)	#0	Robotic locomotor exoskeleton	Exoskeleton assisted walking	46
Hartigan, 2015 (24)	#0	Robotic locomotor exoskeleton	Powered Exoskeleton + Gait Training	46
Zeilig, 2012 (25)	#3	Potential use	ReWalk exoskeleton walking system	44
Field-Fote, 2011 (26)	#16	Locomotor training	4 Sports Training Methods	42

## 4 Discussion

### 4.1 Summary and interpretation of visual analysis results

A total of 1,502 articles were screened, in which the United States dominated; Kadone, Hideki (13 articles, University of Tsukuba, JAPAN) was the author with the highest number of publications; ARCH PHYS MED REHAB (IF = 4.3) was the most cited journal, and topics included molecular biology, immunology, neurology, sports, among other related areas.

**Figure 8:**
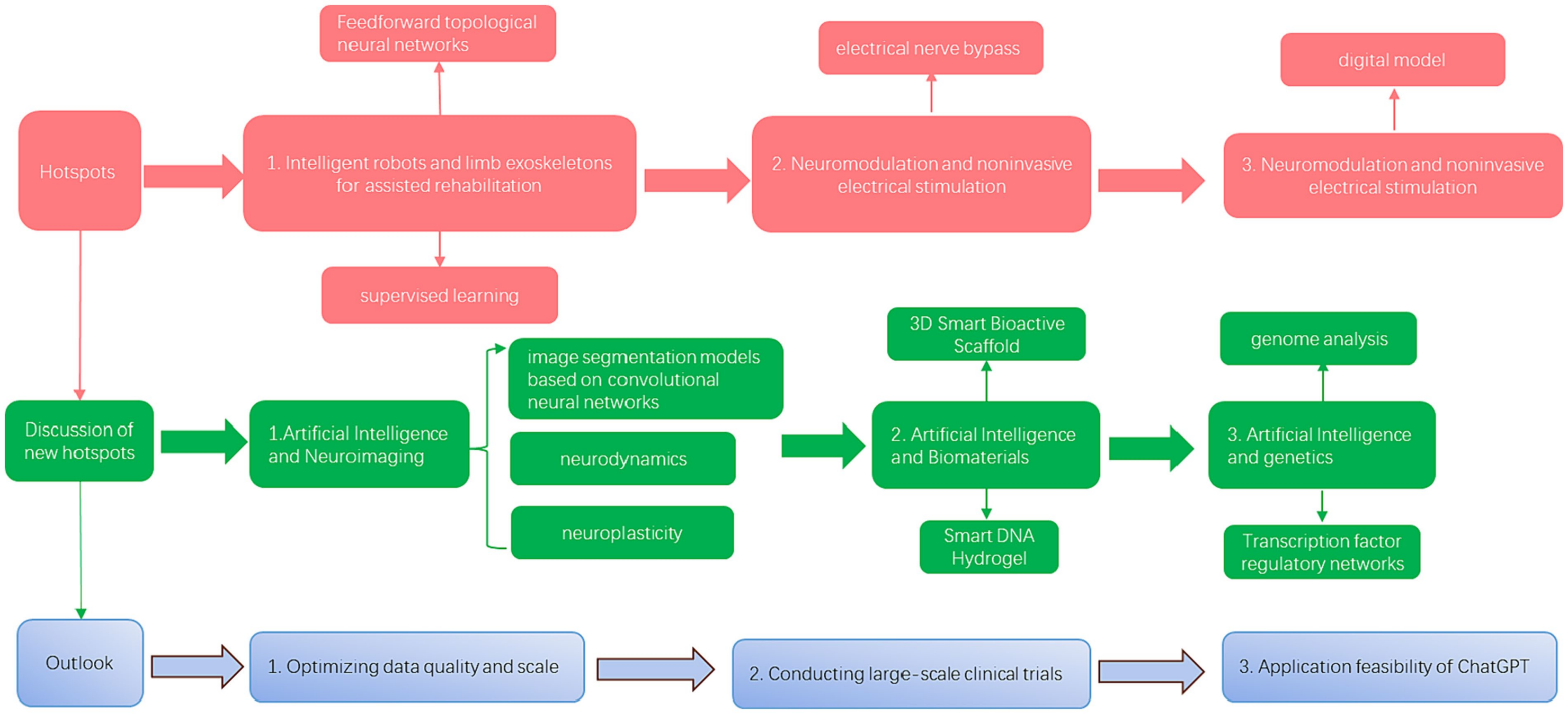
Structure of the discussion segment.

Keyword clustering analysis reveals two main research directions for AI research in spinal cord neural injury and restoration: (1) research on physically biased robot-assisted rehabilitation exercises in AI and (2) research on virtual branches of AI such as deep learning algorithm-assisted brain-computer interfaces and functional electrical stimulation. The results of the keyword breakout analyses show that deep learning and artificial intelligence have been the hottest in the past 5 years (Figure 8).

We performed a co-citation clustering analysis of the included articles to explore the hot directions further and obtained 15 clusters. We performed further analysis to show that the use of artificial intelligence in spinal cord neural injury and restoration focuses on artificial intelligence control electrical stimulation of the spinal cord neuroprosthesis (brain and spinal cord) and information processing. The ultimate goal is to enable patients with paralysis and limb injuries to recover limb function faster through artificially intelligent therapies such as robotic exoskeletons

neuromodulation and brain-computer interfaces.

Next, we performed a co-citation cluster analysis of the literature over the last 5 years. The top research topics in the past 5 years were robotic motion exoskeletons for assisted motor rehabilitation and brain-computer interface and neuromodulation. The field of robotic exoskeleton focuses on three aspects: AI drive, structure, and interface connection; the area of brain-computer interface mainly focuses on functional electrical stimulation + intracortical brain-computer interface technology for the feasibility of clinical neuroprosthesis.

### 4.2 Integration of research hotspots on AI research in spinal cord neural injury and restoration

Based on the above bibliometric results, combined with the authors’ understanding, we identified research hotspots for AI research in spinal cord neural injury and restoration:

1. Intelligent robots and limb exoskeletons for assisted rehabilitation: there is no doubt that these physical branches of AI are the most significant research hotspot of AI in the field of spinal cord neural injury and restoration, which is multiply verified from the keyword analysis, literature co-citation analysis, and the hotspot analysis of highly co-cited literature. Rehabilitation robots are interactive motorized devices that allow fine limb movement and precise measurements (27). They are typically divided into exoskeletons that assist in limb movement by controlling the displacement of each segment and end-effector devices capable of mobilizing the limb from a distal point of application. Sanchez (10) concluded that the field of machine exoskeletons focuses on three aspects: actuation, structure, and interface connectivity. From the above keyword analysis, it can be seen that with time, intelligent robots with deep learning will gradually replace ordinary machine exoskeletons as the emerging hotspot in this field. The authors believe that researchers should vigorously develop various artificial intelligence models, such as feed-forward topological neural networks and supervised learning (8, 28), to improve the safety, tolerance, and walking functional efficacy of robotic exoskeletons to satisfy the needs of clinical patients for more efficient and high-quality treatments.
2. Brain-computer interfaces: a cluster analysis of literature co-citations and co-citations over the last 5 years shows that brain-computer interfaces with deep learning algorithms are one of the continuing hotspots in this field. Brain-computer interface devices are designed to restore lost function and can be used to form electronic “neural bypasses” to circumvent damaged pathways in the nervous system (29, 30). Artificial intelligence techniques applied to brain-computer interfaces can enable disabled and mobility-impaired people to control machines or other devices. Through implanted intracortical brain-computer interfaces, the patient’s cortical signals can be used to direct limb movements (31). For example, Collinger (13) implanted two 96-channel intracortical microelectrodes in a patient’s motor cortex and tested that quadriplegic patients could use this brain-computer interface to achieve neural control of high-performance prostheses rapidly. In addition, Ajiboye (15) restored limb movement in paralyzed patients through an implanted functional electrical stimulation component and an intracortical brain-computer interface. The authors concluded that neuroelectrical stimulation and intracortical brain-computer interface techniques could be combined to restore the neurophysiologic and motor status of SCI patients more effectively. In addition, in the future, researchers could apply machine learning algorithms to decode neuronal activity and control the activation of nerves and muscles in SCI patients with a customized, high-resolution neuromuscular electrical stimulation system, empowering patients with the critical ability to manipulate and release objects.
3. Neuromodulation and noninvasive electrical stimulation: the cluster analysis of literature co-citations and literature co-citations in the last 5 years shows that neuromodulation and noninvasive electrical stimulation are continuing hotspots in this field. Neuroelectrical stimulation is a noninvasive stimulation strategy (32) that transforms neuronal networks from dormant to functional, thereby gradually restoring control over paralyzed muscles (33, 34). In this regard, “numerical models” enhanced by deep learning algorithms are the basis for theoretical simulations of neurostimulation techniques and provide technical guidance for clinical applications. Alexandre Boutet (35) constructed a machine-learning model using fMRI patterns of patients that predicts optimal versus non-optimal settings and has *a priori* clinically optimized DBS (88% accuracy). The authors suggest that future neuroelectrical stimulation research could incorporate deep learning algorithms, such as convolutional neural networks, and use various strategies to neuromodulate the physiological state of the nerves and restore motor function in paralyzed patients. In addition, finding more targeted neuroelectrical stimulation techniques by performing a series of spatially selective stimulations may be one of the future directions.

In summary, our results are relatively reliable based on the bibliometric results and the authors’ understanding.

### 4.3 Experts’ discussion on new research hotspots

In recent years, breakthroughs have been made for AI research in spinal cord neural injury and restoration, positively impacting clinical care. Firstly, artificial intelligence is widely used in neural imaging. For example, image segmentation models based on convolutional neural networks can make excellent contributions to imaging parameters, disease classification, and diagnosis of spinal cord neural injury patients before and after surgery (36, 37). Second, AI can track and analyze in real-time all neural components of various nervous systems, i.e., neural structure, neurodynamics, neuroplasticity, and neural memory (38).

In addition, AI has many applications in repairing spinal cord nerve injuries using biomaterial technology (39). Transplantation of stem cells to the site of injury is a promising approach. Still, it faces many challenges and is highly dependent on the microenvironment provided by the lesion site and the delivery material (7). Using AI to fabricate polymeric biomaterials can provide the microenvironment required for neural stem cell-derived neural network organization to facilitate neural remodeling and repair (40). For example, Li (39) designed a 3D bioactive scaffold and demonstrated that neural network tissues derived from neural stem cells modified by pro-myosin receptor kinase C had strong viability within the scaffold. In addition, Yuan (41) designed DNA hydrogel with extremely high permeability properties by artificial intelligence for repairing a 2-mm spinal cord gap in rats and implanted the proliferation and differentiation of endogenous stem cells to form a nascent neural network. The authors concluded that neural network organization formed by transplantation in 3D innovative bioactive scaffolds may represent a valuable therapy for studying and developing SCI. Still, this technology has not yet been studied on a large scale, and future development should focus on this direction.

Research in the field of genetics: genomic data have high complexity and dimensionality due to differences in genetic structure and functional gene diversity. It is difficult to reveal the sequence patterns and biological mechanisms of genomes using classical analysis methods. At the same time, AI can mine critical biological information from massive multidimensional data, so they are widely used in genome analysis for various diseases (42–44). For example, Artificial intelligence can also discern which gene and signaling pathway is critical for nerve recovery. However, in the field of AI-assisted repair of spinal cord neural injury, the study of genomics and other mechanisms is fragile. In the future, various machine learning techniques, such as AI survival prediction tools, transcription factor regulatory networks, etc., can be utilized to conduct studies related to regeneration-related gene up-regulation and axon growth structural protein production.

### 4.4 Limitations of the study

The WOS core database was searched in this study, and no other English databases were searched. Only WOS data can be analyzed for journal and literature co-citation analysis (a core bibliometrics technique). There is no doubt that WOS, as an authoritative mainstream database, still contains comprehensive and reliable data. Secondly, due to the limitation of the length of the article, this paper does not fully present the details of the specific research methodology in the selected literature but only provides an overview of the ideas in the literature.

### 4.5 Outlook

The following research themes are crucial for future AI research in spinal cord neural injury and restoration.

1. Optimizing data quality and scale: Training AI models require larger, high-quality data pools, and when conducting biomedical explorations, it also requires innovative experimental means to collect relevant data sets.
2. Conducting large-scale clinical trials: Conducting large-scale clinical studies research on AI in spinal cord neural injury and restoration lacks substantial and high-quality clinical trials; therefore, high-quality multicenter and randomized controlled clinical trials should be conducted in the future for in-depth research.
3. Application feasibility of ChatGPT: ChatGPT has recently become a hot topic of discussion, and diagnosing diseases and providing therapeutic advice are promising research areas for ChatGPT. Nonetheless, users who lack specialized knowledge may not be able to recognize the authenticity. People should use ChatGPT cautiously, e.g., just for some initial understanding of the disease.

## 5 Conclusion

This literature metric study reveals dynamic trends in publication patterns and research hotspots for AI-assisted neural injury and restoration of spinal cord neural injuries across the globe. In addition, it identifies potential partners and institutions, major research hotspots, and upcoming research directions in the fields, thereby providing precious guidance for future studies in this area. Finally, the results of this study will be a valuable resource for clinical practitioners, researchers, industrial collaborators, and other interested stakeholders.

## Data availability statement

The original contributions presented in the study are included in the article/Supplementary material, further inquiries can be directed to the corresponding author.

## Author contributions

BY: Writing – review & editing, Writing – original draft, Visualization, Validation, Supervision, Software, Resources, Project administration, Methodology, Investigation, Funding acquisition, Formal analysis, Data curation, Conceptualization. GT: Writing – review & editing, Writing – original draft, Visualization, Validation, Supervision, Software, Resources, Project administration, Methodology, Investigation, Formal analysis, Data curation, Conceptualization. SY: Writing – review & editing, Software, Methodology, Investigation, Data curation, Conceptualization. JX: Writing – review & editing, Software, Methodology, Investigation, Data curation, Conceptualization. LW: Writing – review & editing, Software, Methodology, Investigation, Data curation, Conceptualization.

## Supplementary material

The Supplementary material for this article can be found online at:

https://www.frontiersin.org/articles/10.3389/fneur.2024.1361235/full#supplementary-material

## References

[1] Bican O Minagar A Pruitt AA. The spinal cord: a review of functional neuroanatomy. Neurol Clin. (2013) 31:1–18. doi: 10.1016/j.ncl.2012.09.009 23186894

[2] Guérout N . Plasticity of the injured spinal cord. Cells. (2021) 10:1886. doi: 10.3390/cells10081886, PMID: 34440655 PMC8395000

[3] Luu DK Nguyen AT Jiang M Xu J Drealan MW Cheng J . Deep learning-based approaches for decoding motor intent from peripheral nerve signals. Front Neurosci. (2021) 15:667907. doi: 10.3389/fnins.2021.667907, PMID: 34248481 PMC8260935

[4] Luu DK Nguyen AT Jiang M Drealan MW Xu J Wu T . Artificial intelligence enables real-time and intuitive control of prostheses via nerve Interface. IEEE Trans Biomed Eng. (2022) 69:3051–63. doi: 10.1109/tbme.2022.3160618, PMID: 35302937

[5] Romeo-Guitart D Forés J Herrando-Grabulosa M Valls R Leiva-Rodríguez T Galea E . Neuroprotective drug for nerve trauma revealed using artificial intelligence. Sci Rep. (2018) 8:1879. doi: 10.1038/s41598-018-19767-3, PMID: 29382857 PMC5790005

[6] Guo Y Sun L Zhong W Zhang N Zhao Z Tian W. Artificial intelligence-assisted repair of peripheral nerve injury: a new research hotspot and associated challenges. Neural Regen Res. (2024) 19:663–70. doi: 10.4103/1673-5374.380909, PMID: 37721299 PMC10581578

[7] Liu DD He JQ Sinha R Eastman AE Toland AM Morri M . Purification and characterization of human neural stem and progenitor cells. Cell. (2023) 186:1179–94.e15. doi: 10.1016/j.cell.2023.02.017, PMID: 36931245 PMC10409303

[8] Gorre N Carranza E Fuhrman J Li H Madduri RK Giger M . Midrc Crp10 Ai Interface-an integrated tool for exploring, testing and visualization of Ai models. Phys Med Biol. (2023) 68:074002. doi: 10.1088/1361-6560/acb754, PMID: 36716497 PMC10155272

[9] Evans N Hartigan C Kandilakis C Pharo E Clesson I. Acute cardiorespiratory and metabolic responses during exoskeleton-assisted walking Overground among persons with chronic spinal cord injury. Top Spinal Cord Inj Rehabil. (2015) 21:122–32. doi: 10.1310/sci2102-122, PMID: 26364281 PMC4568093

[10] Sanchez-Villamañan MDC Gonzalez-Vargas J Torricelli D Moreno JC Pons JL. Compliant lower limb exoskeletons: a comprehensive review on mechanical design principles. J Neuroeng Rehabil. (2019) 16:55. doi: 10.1186/s12984-019-0517-9, PMID: 31072370 PMC6506961

[11] Banala SK Kim SH Agrawal SK Scholz JP. Robot assisted gait training with active leg exoskeleton (Alex). IEEE Trans Neural Syst Rehabil Eng. (2009) 17:2–8. doi: 10.1109/tnsre.2008.2008280, PMID: 19211317

[12] Fang CY Tsai JL Li GS Lien AS Chang YJ. Effects of robot-assisted gait training in individuals with spinal cord injury: a Meta-analysis. Biomed Res Int. (2020) 2020:2102785. doi: 10.1155/2020/2102785, PMID: 32280681 PMC7115057

[13] Collinger JL Wodlinger B Downey JE Wang W Tyler-Kabara EC Weber DJ . High-performance Neuroprosthetic control by an individual with tetraplegia. Lancet. (2013) 381:557–64. doi: 10.1016/s0140-6736(12)61816-9, PMID: 23253623 PMC3641862

[14] Angeli CA Edgerton VR Gerasimenko YP Harkema SJ. Altering spinal cord excitability enables voluntary movements after chronic complete paralysis in humans. Brain. (2014) 137:1394–409. doi: 10.1093/brain/awu038, PMID: 24713270 PMC3999714

[15] Ajiboye AB Willett FR Young DR Memberg WD Murphy BA Miller JP . Restoration of reaching and grasping movements through brain-controlled muscle stimulation in a person with tetraplegia: a proof-of-concept demonstration. Lancet. (2017) 389:1821–30. doi: 10.1016/s0140-6736(17)30601-3, PMID: 28363483 PMC5516547

[16] Gill ML Grahn PJ Calvert JS Linde MB Lavrov IA Strommen JA . Neuromodulation of lumbosacral spinal networks enables independent stepping after complete paraplegia. Nat Med. (2018) 24:1677–82. doi: 10.1038/s41591-018-0175-7 30250140

[17] Miller LE Zimmermann AK Herbert WG. Clinical effectiveness and safety of powered exoskeleton-assisted walking in patients with spinal cord injury: systematic review with Meta-analysis. Med Devices (Auckl). (2016) 9:455–66. doi: 10.2147/mder.S103102, PMID: 27042146 PMC4809334

[18] Esquenazi A Talaty M Packel A Saulino M. The Rewalk powered exoskeleton to restore ambulatory function to individuals with thoracic-level motor-complete spinal cord injury. Am J Phys Med Rehabil. (2012) 91:911–21. doi: 10.1097/PHM.0b013e318269d9a3, PMID: 23085703

[19] Louie DR Eng JJ Lam T. Gait speed using powered robotic exoskeletons after spinal cord injury: a systematic review and correlational study. J Neuroeng Rehabil. (2015) 12:82. doi: 10.1186/s12984-015-0074-9, PMID: 26463355 PMC4604762

[20] Wirz M Zemon DH Rupp R Scheel A Colombo G Dietz V . Effectiveness of automated locomotor training in patients with chronic incomplete spinal cord injury: a multicenter trial. Arch Phys Med Rehabil. (2005) 86:672–80. doi: 10.1016/j.apmr.2004.08.004, PMID: 15827916

[21] Bach Baunsgaard C Vig Nissen U Katrin Brust A Frotzler A Ribeill C Kalke YB . Gait training after spinal cord injury: safety, feasibility and gait function following 8 weeks of training with the exoskeletons from Ekso bionics. Spinal Cord. (2018) 56:106–16. doi: 10.1038/s41393-017-0013-7, PMID: 29105657

[22] Nam KY Kim HJ Kwon BS Park JW Lee HJ Yoo A. Robot-assisted gait training (Lokomat) improves walking function and activity in people with spinal cord injury: a systematic review. J Neuroeng Rehabil. (2017) 14:24. doi: 10.1186/s12984-017-0232-3, PMID: 28330471 PMC5363005

[23] Kozlowski AJ Bryce TN Dijkers MP. Time and effort required by persons with spinal cord injury to learn to use a powered exoskeleton for assisted walking. Top Spinal Cord Inj Rehabil. (2015) 21:110–21. doi: 10.1310/sci2102-110, PMID: 26364280 PMC4568092

[24] Hartigan C Kandilakis C Dalley S Clausen M Wilson E Morrison S . Mobility outcomes following five training sessions with a powered exoskeleton. Top Spinal Cord Inj Rehabil. (2015) 21:93–9. doi: 10.1310/sci2102-93, PMID: 26364278 PMC4568090

[25] Zeilig G Weingarden H Zwecker M Dudkiewicz I Bloch A Esquenazi A. Safety and tolerance of the Rewalk™ exoskeleton suit for ambulation by people with complete spinal cord injury: a pilot study. J Spinal Cord Med. (2012) 35:96–101. doi: 10.1179/2045772312y.0000000003, PMID: 22333043 PMC3304563

[26] Field-Fote EC Roach KE. Influence of a locomotor training approach on walking speed and distance in people with chronic spinal cord injury: a randomized clinical trial. Phys Ther. (2011) 91:48–60. doi: 10.2522/ptj.20090359, PMID: 21051593 PMC3017322

[27] Wu J Liu Y Zhao J Zang X Guan Y. Research on theory and a performance analysis of an innovative rehabilitation robot. Sensors (Basel). (2022) 22:3929. doi: 10.3390/s22103929, PMID: 35632338 PMC9147418

[28] Jiang Y Wang Y Miao Z Na J Zhao Z Yang C. Composite-learning-based adaptive neural control for dual-arm robots with relative motion. IEEE Trans Neural Netw Learn Syst. (2022) 33:1010–21. doi: 10.1109/tnnls.2020.3037795, PMID: 33361000

[29] Luo S Rabbani Q Crone NE. Brain-computer Interface: applications to speech decoding and synthesis to augment communication. Neurotherapeutics. (2022) 19:263–73. doi: 10.1007/s13311-022-01190-2, PMID: 35099768 PMC9130409

[30] Cajigas I Vedantam A. Brain-computer Interface, neuromodulation, and Neurorehabilitation strategies for spinal cord injury. Neurosurg Clin N Am. (2021) 32:407–17. doi: 10.1016/j.nec.2021.03.012, PMID: 34053728

[31] Colucci A Vermehren M Cavallo A Angerhöfer C Peekhaus N Zollo L . Brain-computer Interface-controlled exoskeletons in clinical Neurorehabilitation: ready or not? Neurorehabil Neural Repair. (2022) 36:747–56. doi: 10.1177/15459683221138751, PMID: 36426541 PMC9720703

[32] Li X Zhang T Li C Xu W Guan Y Li X . Electrical stimulation accelerates Wallerian degeneration and promotes nerve regeneration after sciatic nerve injury. Glia. (2023) 71:758–74. doi: 10.1002/glia.24309, PMID: 36484493

[33] Dolbow DR Gorgey AS Johnston TE Bersch I. Electrical stimulation exercise for people with spinal cord injury: a healthcare provider perspective. J Clin Med. (2023) 12:3150. doi: 10.3390/jcm12093150, PMID: 37176591 PMC10179213

[34] Barra B Conti S Perich MG Zhuang K Schiavone G Fallegger F . Epidural electrical stimulation of the cervical dorsal roots restores voluntary upper limb control in paralyzed monkeys. Nat Neurosci. (2022) 25:924–34. doi: 10.1038/s41593-022-01106-5, PMID: 35773543

[35] Boutet A Madhavan R Elias GJB Joel SE Gramer R Ranjan M . Predicting optimal deep brain stimulation parameters for Parkinson's disease using functional Mri and machine learning. Nat Commun. (2021) 12:3043. doi: 10.1038/s41467-021-23311-9, PMID: 34031407 PMC8144408

[36] Carson T Ghoshal G Cornwall GB Tobias R Schwartz DG Foley KT. Artificial intelligence-enabled, real-time intraoperative ultrasound imaging of neural structures within the psoas: validation in a porcine spine model. Spine (Phila Pa 1976). (2021) 46:E146–52. doi: 10.1097/brs.0000000000003704, PMID: 33399436 PMC7787186

[37] Nozawa K Maki S Furuya T Okimatsu S Inoue T Yunde A . Magnetic resonance image segmentation of the compressed spinal cord in patients with degenerative cervical myelopathy using convolutional neural networks. Int J Comput Assist Radiol Surg. (2023) 18:45–54. doi: 10.1007/s11548-022-02783-0, PMID: 36342593

[38] Srisuchinnawong A Homchanthanakul J Manoonpong P. Neurovis: real-time neural information measurement and visualization of embodied neural systems. Front Neural Circuits. (2021) 15:743101. doi: 10.3389/fncir.2021.743101, PMID: 35027885 PMC8751631

[39] Li G Zhang B Sun JH Shi LY Huang MY Huang LJ . An Nt-3-releasing bioscaffold supports the formation of Trkc-modified neural stem cell-derived neural network tissue with efficacy in repairing spinal cord injury. Bioact Mater. (2021) 6:3766–81. doi: 10.1016/j.bioactmat.2021.03.036, PMID: 33898877 PMC8044869

[40] Shende P Devlekar NP. A review on the role of artificial intelligence in stem cell therapy: an initiative for modern medicines. Curr Pharm Biotechnol. (2021) 22:1156–63. doi: 10.2174/1389201021666201007122524, PMID: 33030129

[41] Yuan T Shao Y Zhou X Liu Q Zhu Z Zhou B . Highly permeable DNA supramolecular hydrogel promotes neurogenesis and functional recovery after completely transected spinal cord injury. Adv Mater. (2021) 33:e2102428. doi: 10.1002/adma.202102428, PMID: 34296471

[42] Zhang Z He T Huang L Li J Wang P. Immune gene prognostic signature for disease free survival of gastric Cancer: translational research of an artificial intelligence survival predictive system. Comput Struct Biotechnol J. (2021) 19:2329–46. doi: 10.1016/j.csbj.2021.04.025, PMID: 34025929 PMC8111455

[43] Sarajlic P Plunde O Franco-Cereceda A Bäck M. Artificial intelligence models reveal sex-specific gene expression in aortic valve calcification. JACC Basic Transl Sci. (2021) 6:403–12. doi: 10.1016/j.jacbts.2021.02.005, PMID: 34095631 PMC8165113

[44] He T Huang L Li J Wang P Zhang Z. Potential prognostic immune biomarkers of overall survival in ovarian Cancer through comprehensive bioinformatics analysis: a novel artificial intelligence survival prediction system. Front Med (Lausanne). (2021) 8:587496. doi: 10.3389/fmed.2021.587496, PMID: 34109184 PMC8180546

