# Supplementary material for: Global research trends and hotspots of artificial intelligence research in spinal cord neural injury and restoration—a bibliometrics and visualization analysis
Source: Front Neurol. 2024 Apr 2;15:1361235. doi: 10.3389/fneur.2024.1361235 (PMC11018935; doi:10.3389/fneur.2024.1361235)
Supplement: Supplementary file 1 [file Presentation_1.PPTX]

## Slide 1
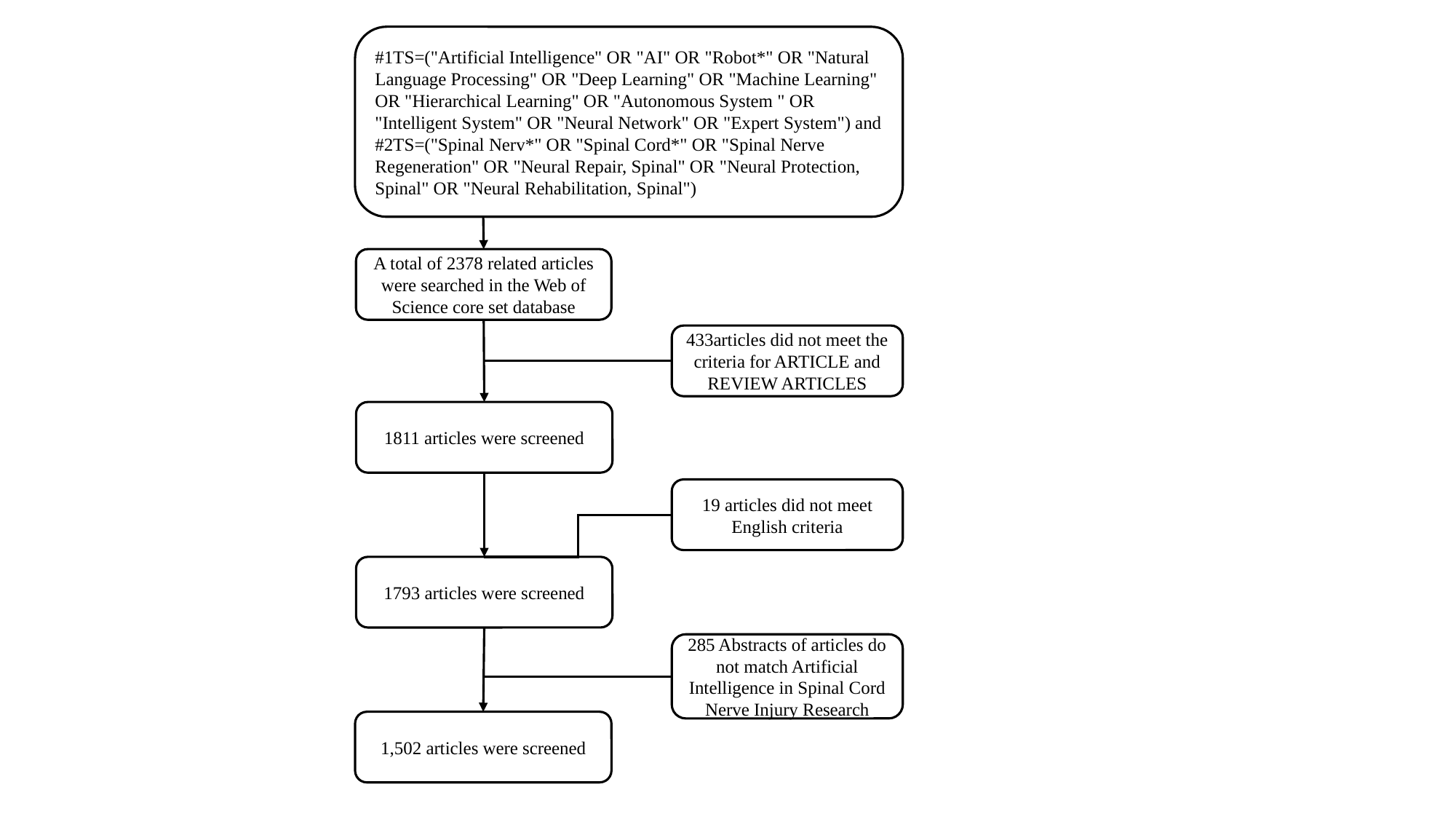

#1TS=("Artificial Intelligence" OR "AI" OR "Robot*" OR "Natural Language Processing" OR "Deep Learning" OR "Machine Learning" OR "Hierarchical Learning" OR "Autonomous System " OR "Intelligent System" OR "Neural Network" OR "Expert System") and #2TS=("Spinal Nerv*" OR "Spinal Cord*" OR "Spinal Nerve Regeneration" OR "Neural Repair, Spinal" OR "Neural Protection, Spinal" OR "Neural Rehabilitation, Spinal")
A total of 2378 related articles were searched in the Web of Science core set database
433articles did not meet the criteria for ARTICLE and REVIEW ARTICLES
1811 articles were screened
19 articles did not meet English criteria
1793 articles were screened
285 Abstracts of articles do not match Artificial Intelligence in Spinal Cord Nerve Injury Research
1,502 articles were screened

## Slide 2
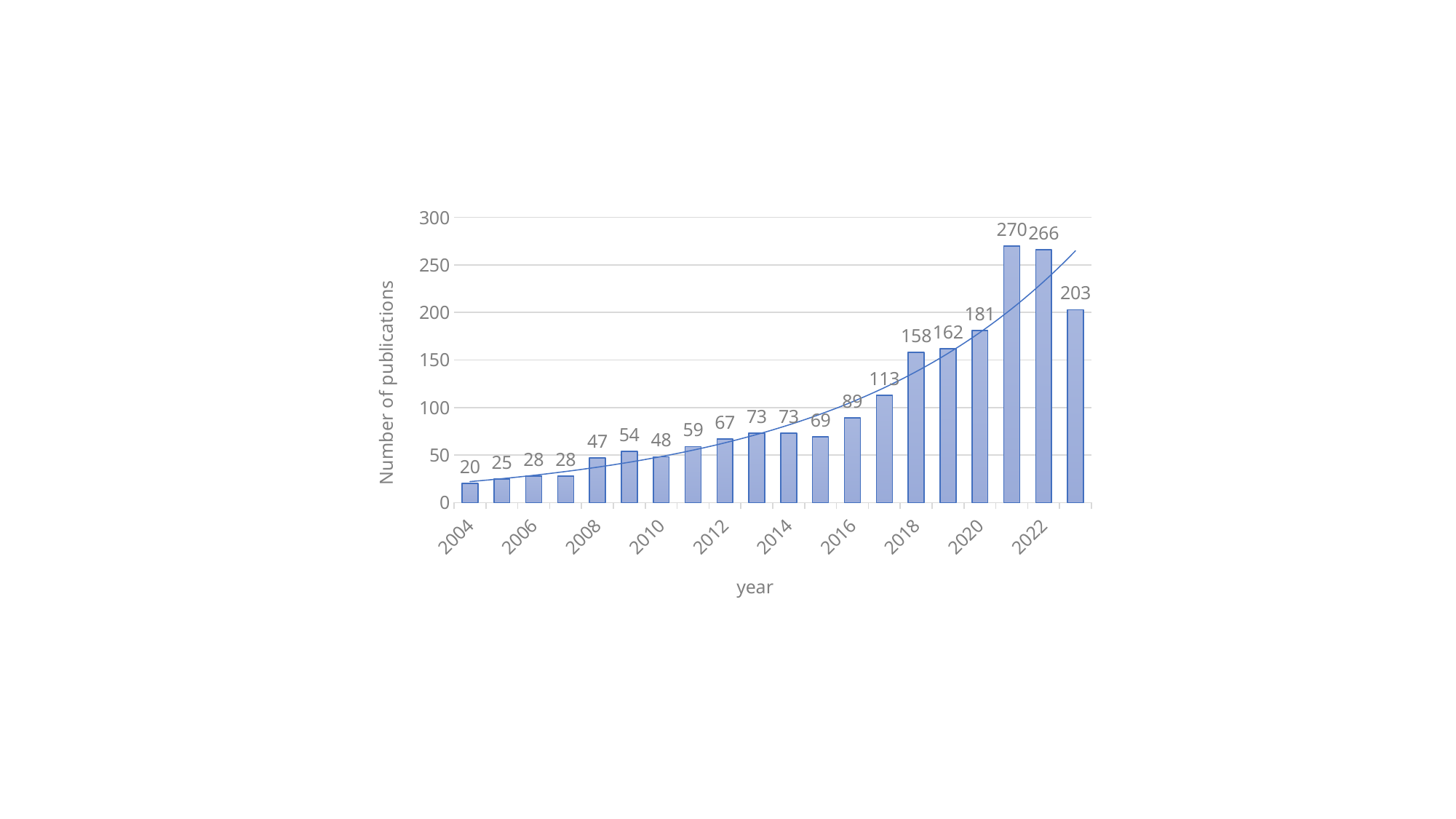

### Chart
| Category | |
|---|---|
| 2004 | 20.0 |
| 2005 | 25.0 |
| 2006 | 28.0 |
| 2007 | 28.0 |
| 2008 | 47.0 |
| 2009 | 54.0 |
| 2010 | 48.0 |
| 2011 | 59.0 |
| 2012 | 67.0 |
| 2013 | 73.0 |
| 2014 | 73.0 |
| 2015 | 69.0 |
| 2016 | 89.0 |
| 2017 | 113.0 |
| 2018 | 158.0 |
| 2019 | 162.0 |
| 2020 | 181.0 |
| 2021 | 270.0 |
| 2022 | 266.0 |
| 2023 | 203.0 |

## Slide 3
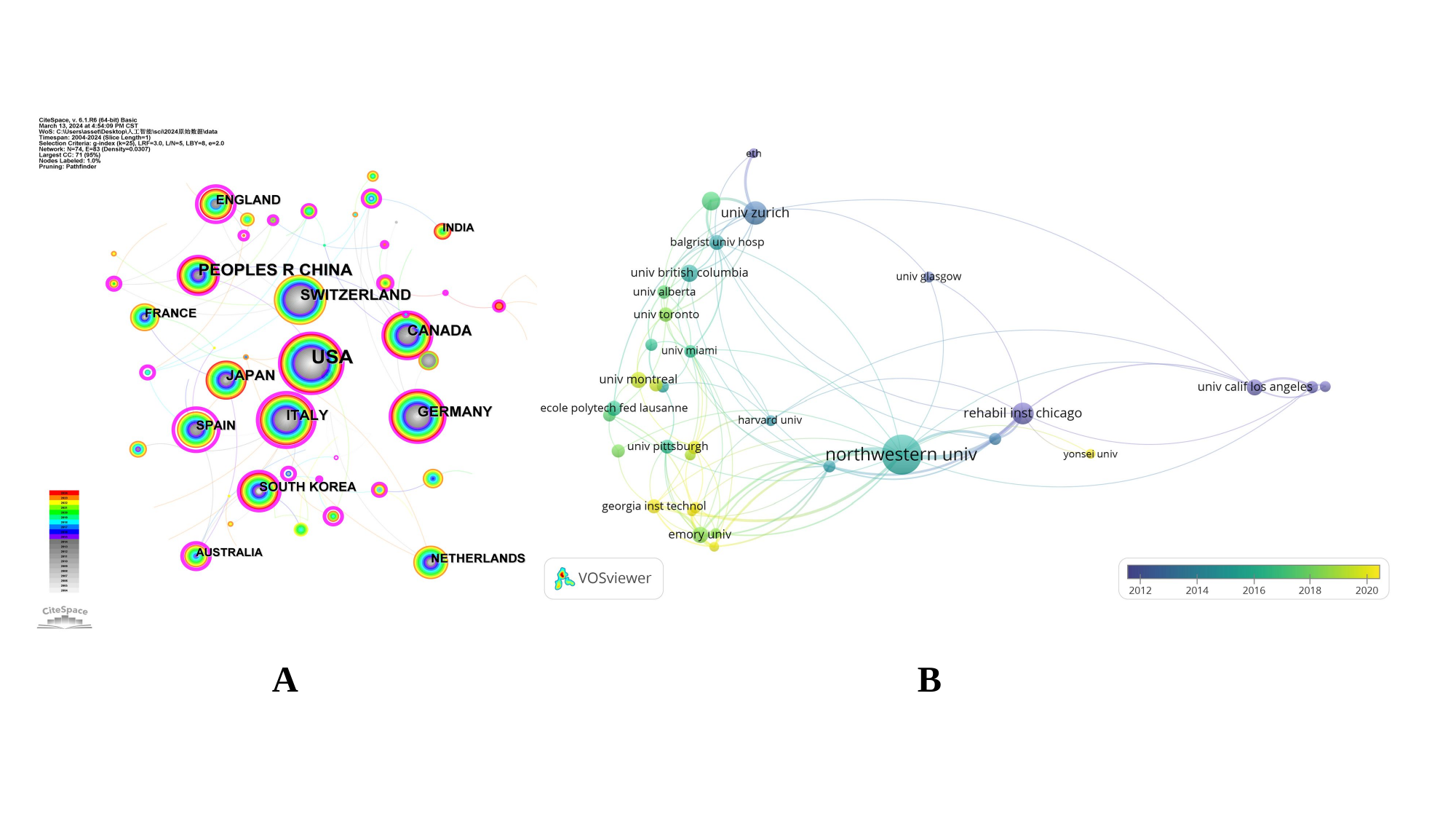

A
B

## Slide 4
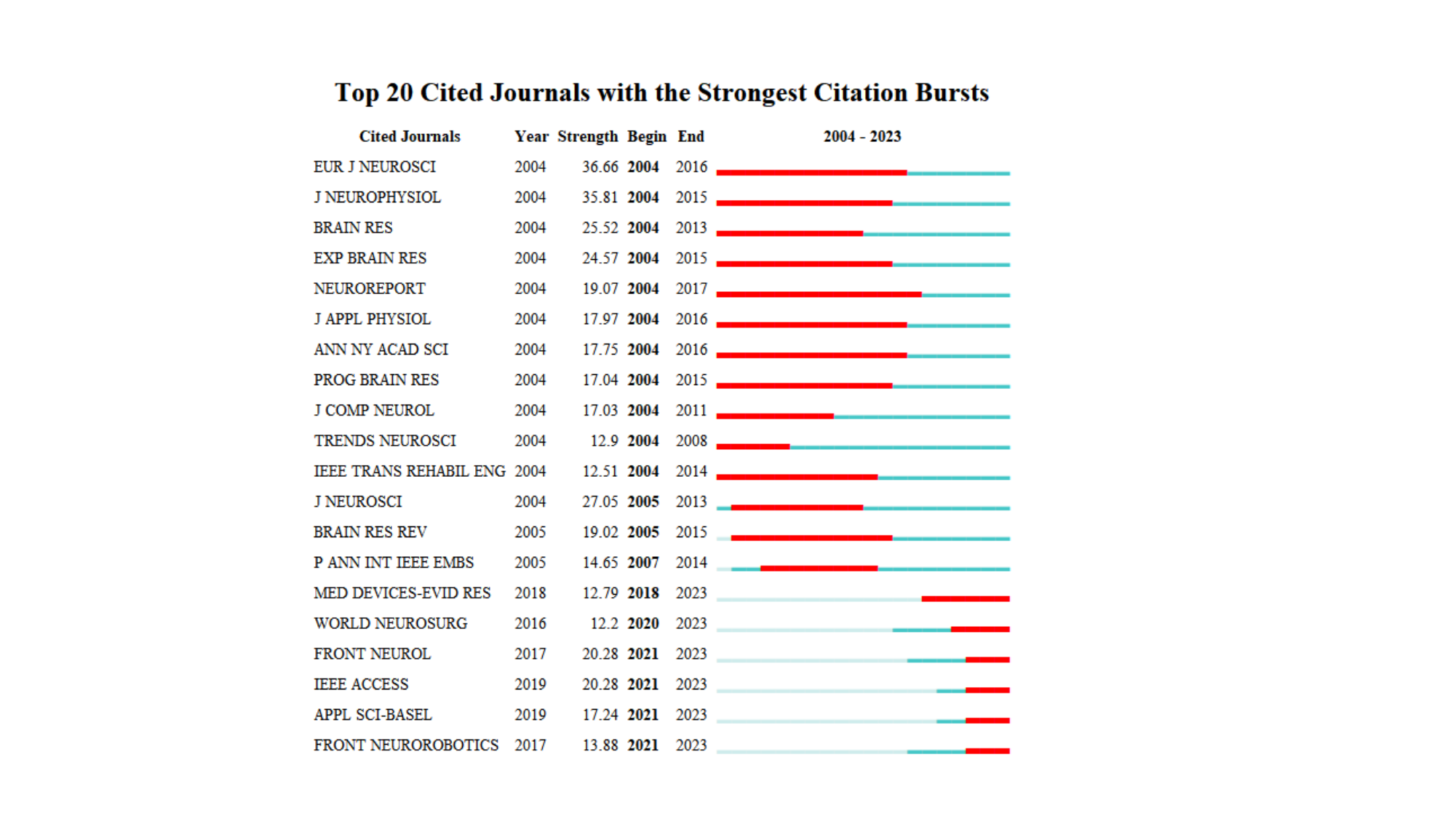

## Slide 5
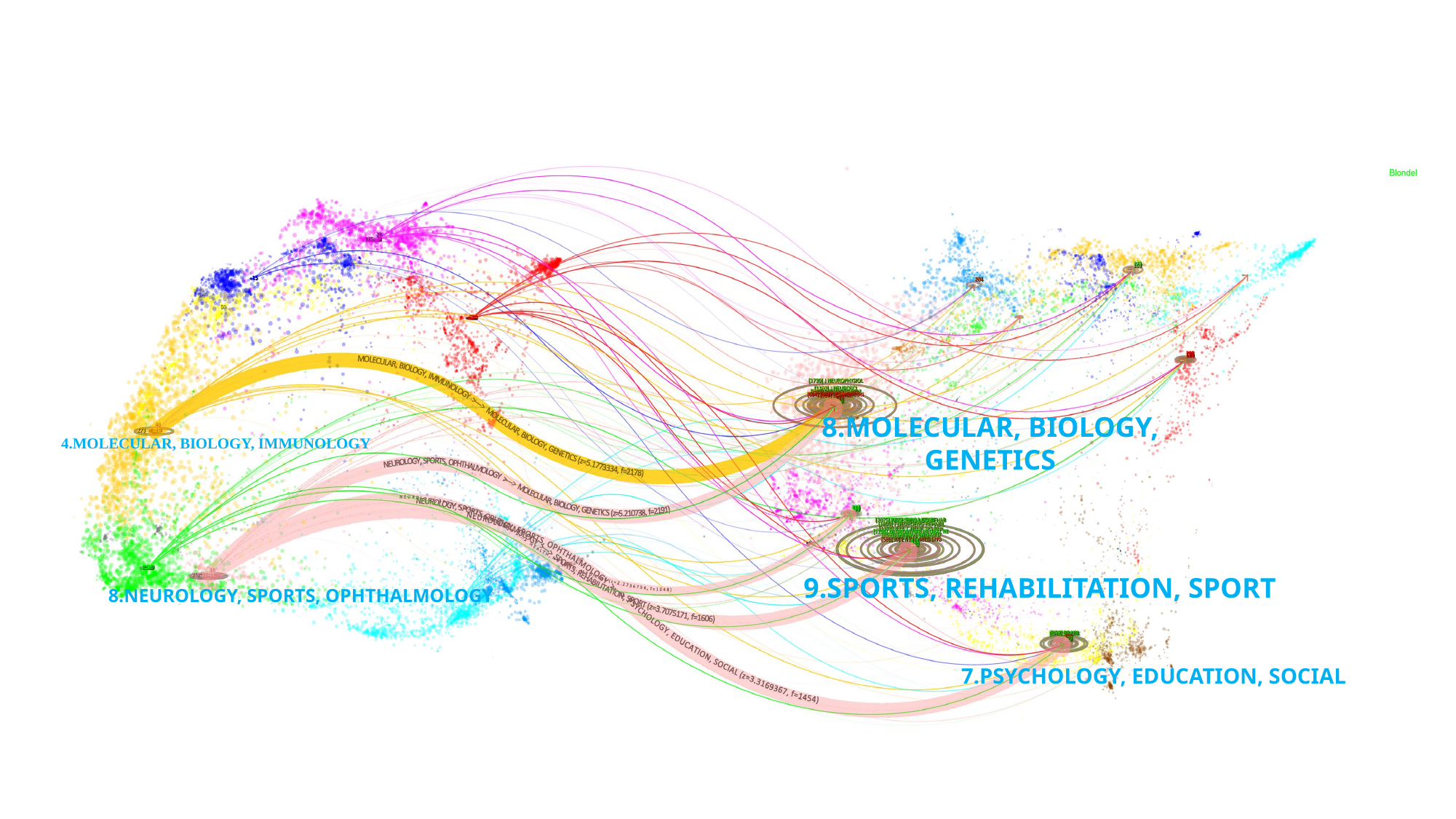

8.MOLECULAR, BIOLOGY, GENETICS
4.MOLECULAR, BIOLOGY, IMMUNOLOGY
9.SPORTS, REHABILITATION, SPORT
8.NEUROLOGY, SPORTS, OPHTHALMOLOGY
7.PSYCHOLOGY, EDUCATION, SOCIAL

## Slide 6
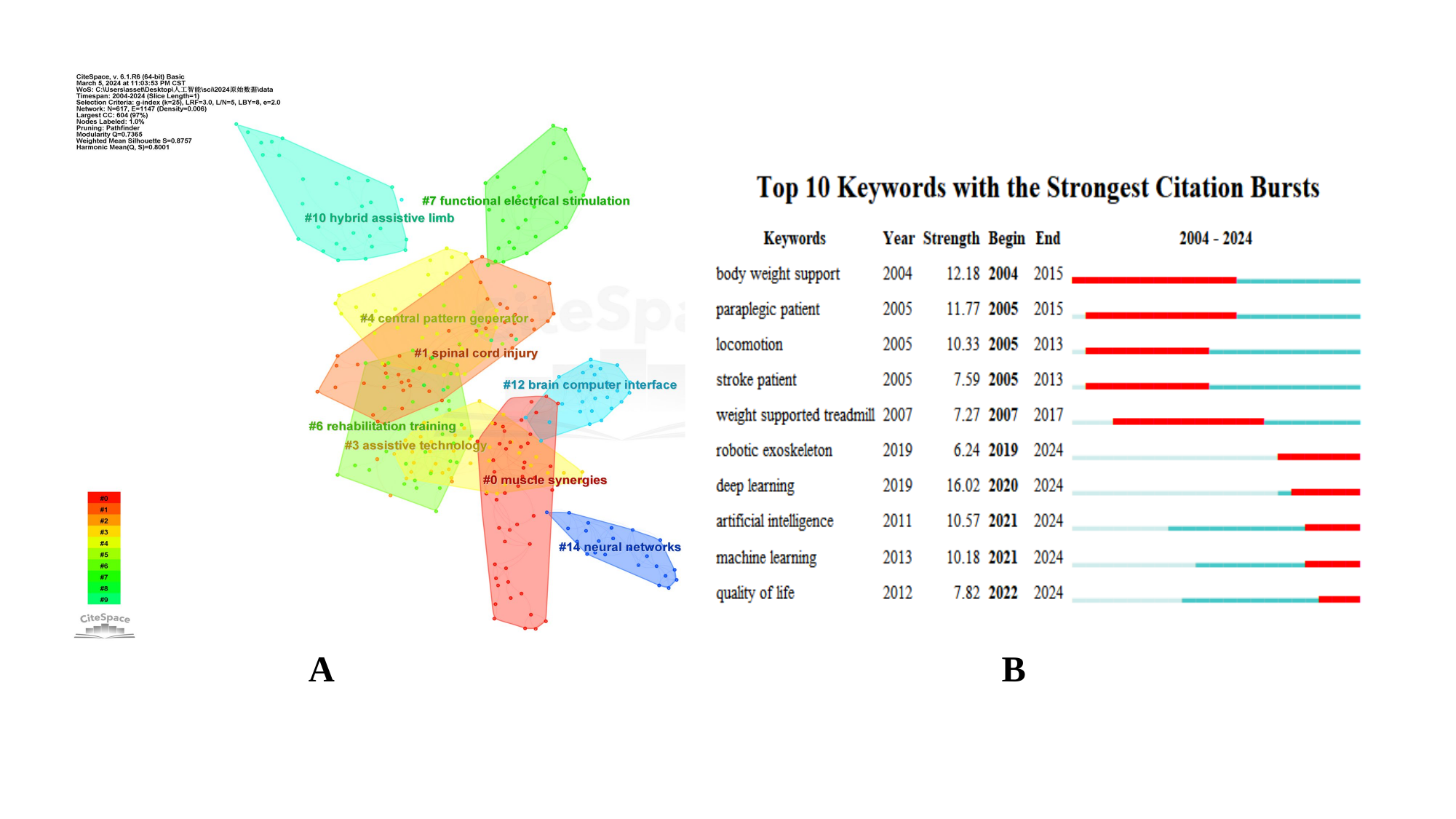

A
B

## Slide 7
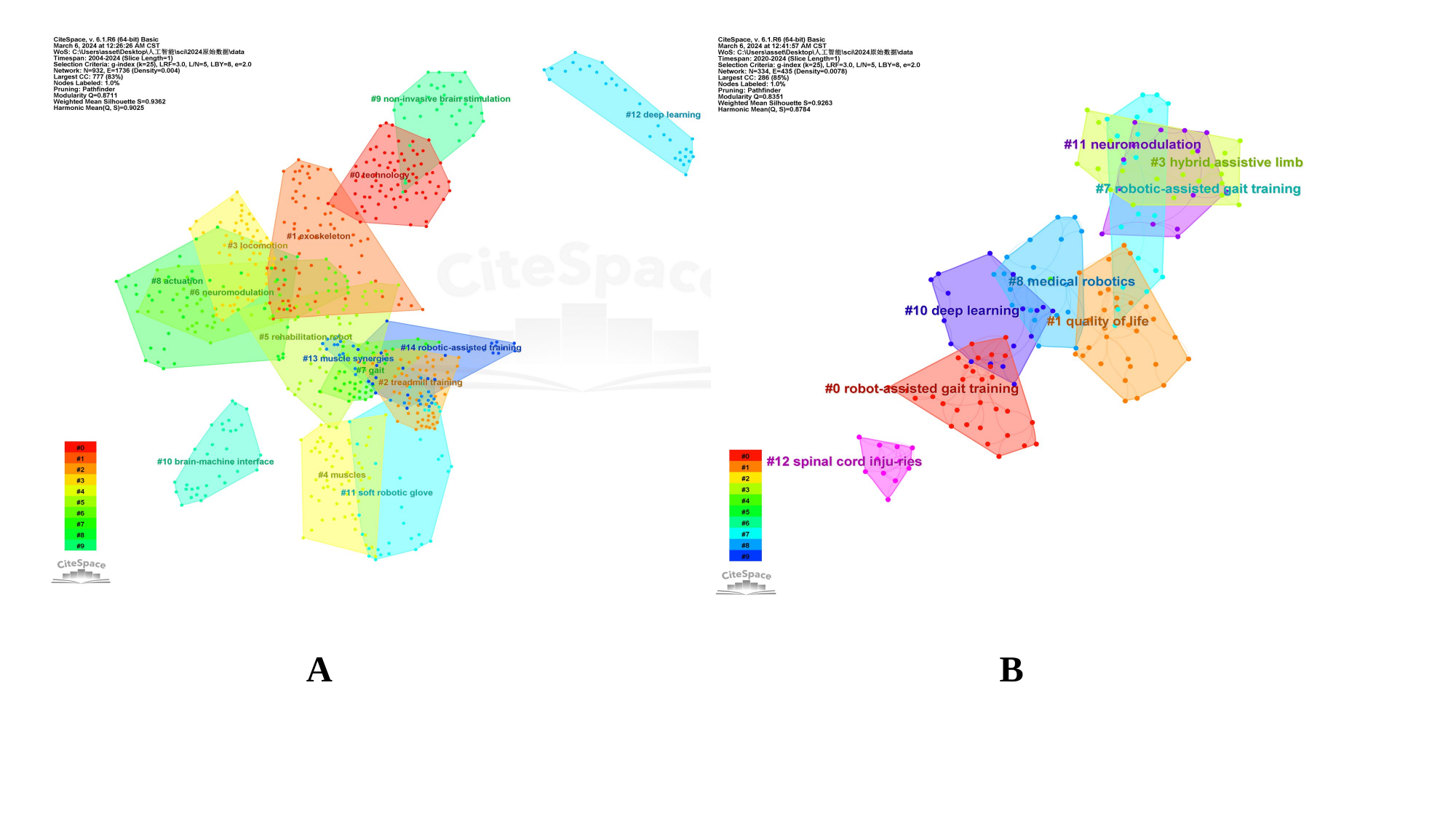

A
B

## Slide 8
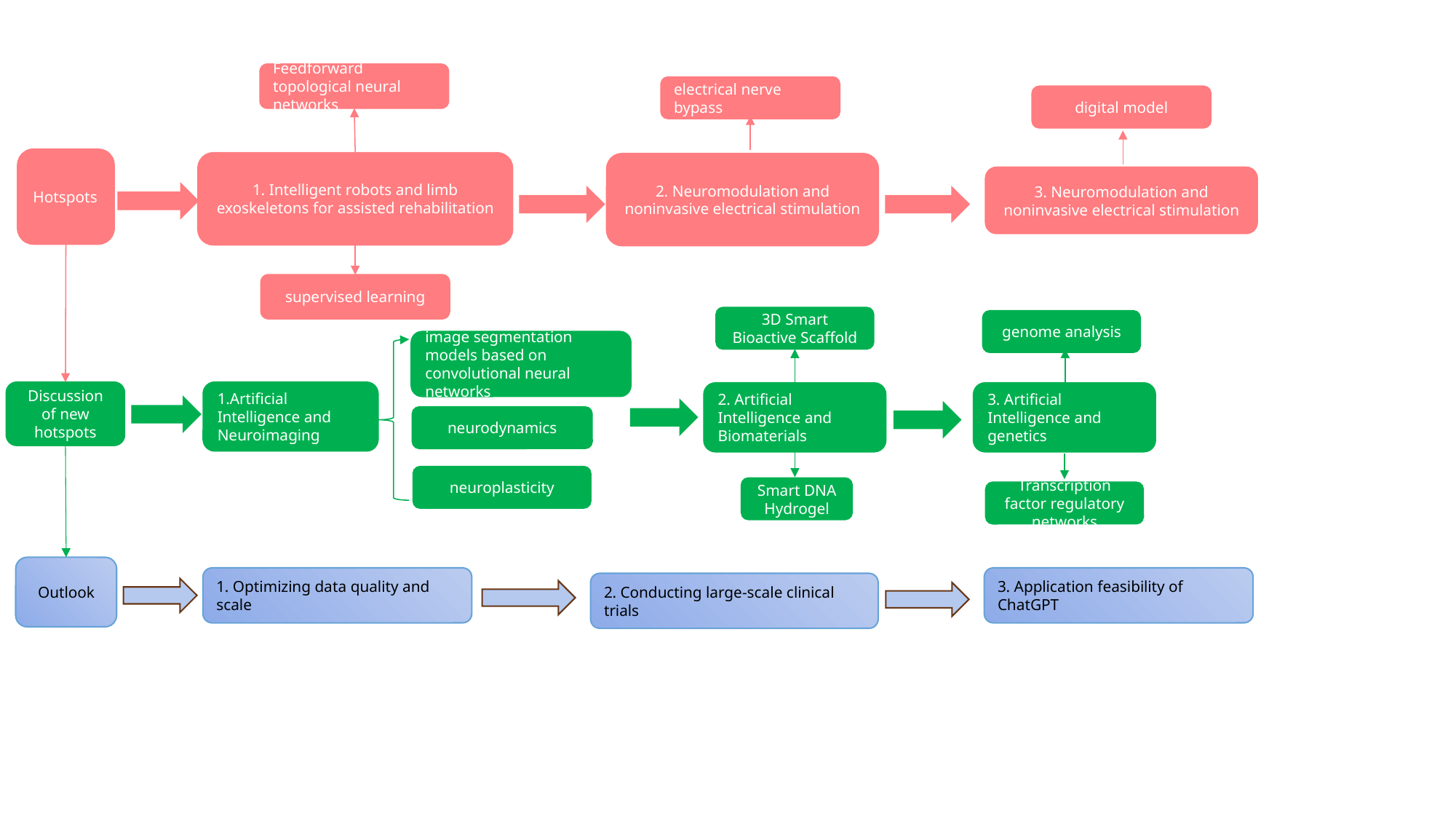

Feedforward topological neural networks
electrical nerve bypass
digital model
Hotspots
1. Intelligent robots and limb exoskeletons for assisted rehabilitation
2. Neuromodulation and noninvasive electrical stimulation
3. Neuromodulation and noninvasive electrical stimulation
supervised learning
3D Smart Bioactive Scaffold
genome analysis
image segmentation models based on convolutional neural networks
1.Artificial Intelligence and Neuroimaging
2. Artificial Intelligence and Biomaterials
3. Artificial Intelligence and genetics
neurodynamics
neuroplasticity
Smart DNA Hydrogel
Transcription factor regulatory networks
Outlook
3. Application feasibility of ChatGPT
1. Optimizing data quality and scale
2. Conducting large-scale clinical trials
Discussion of new hotspots
